# Targeting CEACAM5: Biomarker Characterization and Fluorescent Probe Labeling for Image-Guided Gastric Cancer Surgery

**DOI:** 10.3390/biomedicines13081812

**Published:** 2025-07-24

**Authors:** Serena Martinelli, Sara Peri, Cecilia Anceschi, Anna Laurenzana, Laura Fortuna, Tommaso Mello, Laura Naldi, Giada Marroncini, Jacopo Tricomi, Alessio Biagioni, Amedeo Amedei, Fabio Cianchi

**Affiliations:** 1 Department of Clinical and Experimental Medicine, University of Florence, 50139 Florence, Italy; laura.fortuna@unifi.it (L.F.); amedeo.amedei@unifi.it (A.A.); fabio.cianchi@unifi.it (F.C.); 2 Department of Experimental and Clinical Biomedical Sciences “Mario Serio”, University of Florence, 50139 Florence, Italy; sara.peri@unifi.it (S.P.); cecilia.anceschi@unifi.it (C.A.); anna.laurenzana@unifi.it (A.L.); tommaso.mello@unifi.it (T.M.); laura.naldi@unifi.it (L.N.); alessio.biagioni@unifi.it (A.B.); 3 Biomolecular Diagnostic Laboratories, Via N. Porpora, 50144 Florence, Italy; giada.marroncini@bdmail.it; 4 Department of Chemistry ‘Ugo Schiff’, University of Firenze, Via della Lastruccia 13, 50019 Florence, Italy; jacopo.tricomi@unifi.it; 5 Network of Immunity in Infection, Malignancy and Autoimmunity (NIIMA), Universal Scientific Education and Research Network (USERN), 50139 Florence, Italy

**Keywords:** image-guided surgery, gastric cancer, fluorescent tracers

## Abstract

**Background**: Gastric cancer (GC) is a malignant tumor of the gastrointestinal tract, characterized by high mortality rates and responsible for about one million new cases each year globally. Surgery is the main treatment, but achieving radical resection remains a relevant intraoperative challenge. Fluorescence-guided surgery offers clinicians greater capabilities for real-time detection of tumor nodules and visualization of tumor margins. In this field, the main challenge remains the development of fluorescent dyes that can selectively target tumor tissues. **Methods**: we examined the expression of the most suitable GC markers, including carcinoembryonic antigen cell adhesion molecule-5 (CEACAM5) and Claudin-4 (CLDN4), in GC cell lines. To further evaluate their expression, we performed immunohistochemistry (IHC) on tumor and healthy tissue samples from 30 GC patients who underwent partial gastrectomy at the Digestive System Surgery Unit, AOU Careggi, Florence. Additionally, we validated anti-CEACAM5 expression on patient-derived organoids. Furthermore, we developed a fluorescent molecule targeting CEACAM5 on the surface of GC cells and assessed its binding properties on patient tissue slices and fragments. **Results**: in this work, we first identified CEACAM5 as an optimal GC biomarker, and then we developed a fluorescent antibody specific for CEACAM5. We also evaluated its binding specificity for GC cell lines and patient-derived tumor tissue, achieving an optimal ability to discriminate tumor tissue from healthy mucosa. **Conclusions**: Overall, our results support the development of our fluorescent antibody as a promising tumor-specific imaging agent that, after further in vivo validation, could improve the accuracy of complete tumor resection.

## 1. Introduction

Gastric cancer (GC) is the fifth most common cancer and cause of death worldwide, with over 1 million new cases diagnosed annually [1,2]. Currently, surgery is the primary curative option, and research on surgical quality has become a key area of focus. Distinguishing malignant tissue from healthy mucosa (HM) relies heavily on the surgeon’s tactile and visual assessments, as well as their experience. This dependence increases the risk of positive surgical margins (PSMs) or the number of residual tumors remaining after resection [3]. Additionally, distinguishing tumor-positive lymph nodes from non-tumor lymph nodes during surgery is not yet feasible. This requires extensive lymphadenectomy, thus increasing postoperative complications and morbidity for the patients [4].

NIR (NIR) fluorescence imaging is a real-time approach that utilizes an NIR fluorescent agent in combination with a specialized imaging system. These systems detect light emitted by the fluorescent agent after excitation with a suitable light source. Since the human eye cannot perceive NIR wavelengths, fluorescent agents do not interfere with the standard surgical field. Indocyanine green (ICG) is a diagnostic agent that fluoresces when stimulated by a laser or NIR light (λ ≥ 800 nm). This fluorescence can be integrated into the surgical process using either conventional laparoscopy or precision surgery robotic platforms such as the da Vinci system [5,6]. ICG has a lymphatic tropism that allows it to follow lymphatic vessels and accumulate in lymph nodes when injected into the submucosa or subserosa, aiding its detection [7,8,9,10,11,12]. Using NIR technology, lymphatic vessels and nodes containing ICG can be clearly distinguished from the surrounding fatty tissue, thus making ICG a valuable tool to map lymph nodes and to improve dissection in GC surgical procedures [13,14]. Although it offers several advantages, ICG functions as an untargeted fluorescent contrast agent. Accordingly, the development of a targeted fluorescent contrast agent that can actively accumulate in tumor tissue by recognizing specific biomarkers is critical [15]. Such an agent could precisely delineate tumor margins and visualize metastatic lymph nodes during gastrectomy. Tumor-targeted intraoperative fluorescence imaging could offer surgeons a real-time view of tumor location and extent, achieving significant value in the management of locoregional and metastatic GC, potentially improving patient outcomes [16].

This study aimed to find an optimal GC biomarker to discriminate tumor tissue from healthy tissue, and then to develop a GC-specific molecular imaging probe based on the fluorescent detection of NIR at 800 nm. The designed probe, which we named CMG, is projected to be integrated with surgical imaging platforms, enabling the real-time visualization of cancerous tissues during surgical procedures. By improving tumor delineation, this new probe has the potential to significantly increase the accuracy of fluorescence-guided surgery, minimizing residual disease and improving patient outcomes.

## 2. Materials and Methods

### 2.1. Cell Cultures

AGS GC and NCI-n87 cells were purchased from ATCC (CRL-1739 and CRL-5822, respectively), the 23132/87 cell line [17] (hereafter referred to as ACC-201) was provided by the Leibniz Institute (DSMZ-German Collection of Microorganisms and Cell Cultures), MKN74 was purchased from Cytion, and the HFE145 cell line was kindly provided by Professor Christine Varon. Each cell line was maintained with appropriate media supplemented with 10% FBS (fetal bovine serum), 1% L-glutamine (2 mM), and 1% PS (penicillin 100 U/mL and streptomycin 100 µg/mL): DMEM-F12 for HFE145, F12 Kaighn’s Modification (Corning, Milano, Italy) for AGS, and RPMI (Euroclone, Milano, Italy) for NCI-N87, MKN74, and ACC201. The AGS and ACC-201 cell lines were derived from primary gastric adenocarcinoma patients. NCI-n87 and MKN74 were isolated from the liver metastasis of a gastric carcinoma patient. HFE145 was derived from human normal gastric epithelial cells and then immortalized.

### 2.2. Organoid Culture

GC and adjacent HM biopsies were obtained after a partial gastrectomy at the Digestive System Surgery, AOU Careggi, Florence. Samples were obtained with patients’ informed consent according to Ethics Committee authorization number 27004_bio. The tissues (both tumor and healthy), preserved from the explant in culture medium supplemented with antibiotics (penicillin/streptomycin, ampicillin, kanamycin, amphotericin B, and fluconazole) and 10 µM Y-27632 (Merck Millipore, Milan, Italy) at 4 °C, were processed within 24 h of explantation. The HM fragments were cleaned from the mucus and muscular layer, washed in PBS, and minced into small pieces of 1–5 mm. These pieces were collected in cold chelating buffer (5.6 mM Na_2_HPO_4_, 8 mM KH_2_PO_4_, 96.2 mM NaCl, 1.6 mM KCl, 43.4 mM sucrose, 54.9 mM D-sorbitol, and 0.5 mM DL-dithiotreitol) and washed several times until the supernatant was clear, then incubated in the same chelating buffer containing 2 mM ethylenediaminetetraacetic acid (EDTA) at 4 °C in continuous motion for 30 min. The fragments were then crushed using a slide in order to release the gastric glands. After recovering the glands and washing them in the medium, they were plated in drops of 75% basement membrane extract (BME; Cultrex BME RGF type 2, Bio-Techne), three drops for each 24-well multi-plate. The tumor tissue was instead fragmented and incubated with proteolytic enzymes, such as collagenase II (1.5 mg/mL) and hyaluronidase (20 µg/mL), in Advanced DMEM/F12 supplemented with 1% BSA, antibiotics (penicillin/streptomycin, ampicillin, kanamycin, amphotericin B, and fluconazole) and 10 µM Y-27632 for 30 min at 37 °C with shaking. The material was filtered in a 100 µm strainer (Greiner, Brescia, Italy) and washed with medium twice, and the obtained pellet was plated in drops of 75% BME, as above. Optional lysis of erythrocytes was performed by incubating glands or cell pellets for 5 min with an erythrocyte lysis buffer (Euroclone) on ice and then washed twice with PBS prior to plating in BME. After allowing the BME to polymerize for 15 min at 37 °C in organoid culture medium containing Advanced Dulbecco’s Modified Eagle Medium (DMEM)/F12 (Gibco, Monza, Italy) supplemented with penicillin/streptomycin, 10 mM HEPES, 1% GlutaMAX, 0.5 nM Wnt surrogate-Fc fusion protein (IPA Immunoprecise Antibodies, Utrecht, The Netherlands), 2% RSPO3-Fc fusion protein conditioned medium (IPA Immunoprecise Antibodies, Utrecht, The Netherlands), 1% Noggin-Fc fusion protein conditioned medium (IPA Immunoprecise Antibodies, Utrecht, The Netherlands), 1x Primocin (Invivogen, San Diego, CA, USA), 1% B27-vitamin A (Gibco), 1.25 mM N-acetylcysteine (Sigma-Aldrich, St Louis, MO, USA), 50 ng/mL epidermal growth factor (EGF) (Peprotech, Hamburg, Germany), 200 ng/mL fibroblast growth factor-10 (FGF10) (Peprotech, Hamburg, Germany), 1 nM Gastrin (Tocris, Bristol, UK), 2 µM A-83-01 (Tocris), and 10 µM Rho-associated protein kinase (ROCK) inhibitor Y-27632 (Sigma-Aldrich), the medium was changed every three days, and, after approximately 14 days of culture at 37 °C and 5% CO_2_, the fragments had organized to recreate the original organ, forming spherical structures with a hollow internal lumen. Once the organoids had grown to the confluence of the BME droplet, they were mechanically dissociated with TrypLE™ Express (Gibco) using a pipette, washed in PBS, and replated in matrigel. Organoids that had undergone at least one passage could be used for further experiments.

### 2.3. Western Blotting

AGS, ACC-201, HFE145, NCI-n87, and MKN74 GC cells were lysed in a radioimmunoprecipitation assay (RIPA) lysis buffer (Merck Millipore, Milan, Italy) with the addition of Pierce Protease Inhibitor Tablets (Thermo Fisher Scientific, Monza, Italy) for the isolation of proteins. All passages were carried out on ice, as previously described [18,19]. The protein concentration was measured using the Bradford reagent (Merck Millipore, Roma, Italy), and 40 μg of proteins were loaded on each well in Laemmli buffer, separated on precast Bolt 4–12% Bis-Tris Plus (Invitrogen, Monza, Italy) at the same time as the marker (Page Ruler Plus Prestained Protein Ladder, Thermo Fisher Scientific), and transferred to a polyvinylidene difluoride (PVDF) membrane using the iBlot 2 System (Thermo Fischer Scientific, Milan, Italy). Membranes were incubated for 5 min with the EveryBlot Blocking Buffer (Bio-Rad, Milano, Italy) and then incubated overnight at 4 °C with the 1:1000 diluted antibodies. Primary antibodies against carcinoembryonic antigen 5 (CEACAM5), Claudin4 (CLDN4) (#A12912), PIGR (#A6130), Olfm4 (#A15387), and anti-EpCAM (#A19301) were from AbClonal, Roma, Italy; anti-actin was from Thermo Fisher Scientific. In addition, an anti-CEACAM5 CMG-conjugated antibody was used. The next day, 1 h of incubation at RT with secondary antibodies was performed using the goat anti-rabbit IgG Alexa Fluor 700 antibody (Thermo Fisher Scientific, Italy), and membranes were visualized using the Odyssey Infrared Imaging System (LI-COR^®^ Bioscience, Lincoln, OR, USA). When the CMG-conjugated antibody was used, incubation with the secondary antibody was not performed.

### 2.4. Immunohistochemical Analysis

Formalin-fixed paraffin-embedded tumor fragments were analyzed by immunohistochemistry (IHC) to assess the levels of CEACAM5 and CLDN4.

Following deparaffinization and rehydration, formalin-fixed tissue slices underwent antigen retrieval by boiling in Citrate Buffer (pH 6) at 95 °C for 10 min. To inhibit tissue peroxidase activity, the slices were then treated with a 6% H_2_O_2_ solution for 30 min at room temperature (RT), followed by blocking in PBS containing 2% BSA for 1 h. Next, the tissue sections were incubated overnight at 4 °C with the following primary antibodies: anti-CEACAM5 (#A22266) and anti-CLDN4 (#A12912) from AbClonal. The sections were then incubated with specific secondary antibodies conjugated to horseradish peroxidase (HRP-linked anti-rabbit IgG, #7074, Cell Signaling Technology, USA) for 1 h, after which antigen detection was performed using the SignalStain^®^ DAB Substrate Kit (#8059, Cell Signaling Technology, Danvers, MA, USA). DAB-positive cells were subsequently analyzed and quantified using ImageJ 1.54p software [20] and GraphPad Prism 8.4.3 software.

### 2.5. Immunofluorescence Staining

Staining of slices: Formalin-fixed paraffin-embedded tumor biopsies were analyzed by immunofluorescence to assess the levels of CEACAM5. Deparaffinization, rehydration, and antigen retrieval were performed as described above. The slices were then treated with 0.1 M glycine for 30 min to decrease tissue autofluorescence, then fixed in 10% neutral buffered formalin, permeabilized in 0.05% Triton-X-100, blocked in 2% BSA (bovine serum albumin) in PBS (phosphate buffer saline), and incubated with rabbit monoclonal anti-CEACAM5 (AbClonal, #A22266) and Hoechst 33342 (NucBlue Live Cell Stain Ready Probes reagent, R37605, Invitrogen-Thermo Fisher Scientific, USA) overnight. The next day, sections were washed twice in PBS and stained with the anti-rabbit IgG Alexa Fluor488 secondary antibody for 1 h at RT in the dark. Finally, the slices were washed and mounted in fluorescent Prolong Gold Antifade medium (Invitrogen, Invitrogen, Monza, Italy). Negative control sections (no exposure to the primary antibody) were processed concurrently with the other sections for all immunohistochemical studies. Images were acquired with a Leica Stellaris 5 confocal laser scanning microscope equipped with LAS X software 4.0.2 (Leica Microsystems, Wetzlar, Germany). When the CMG-conjugated antibody was used, incubation with the secondary antibody was not performed, and the signal detection was performed with the Odyssey Infrared Imaging System (LI-COR^®^ Bioscience, USA) at 800 nm.

Organoid staining: The organoids were picked from the matrigel and washed with PBS, fixed with 10% formalin, permeabilized with 0.1% Tween20 in PBS, blocked in 2% BSA and 0.1% Triton-X-100 in PBS, and incubated with rabbit monoclonal anti-CEACAM5 (AbClonal, #A22266) and Hoechst 33342 (NucBlue Live cell Stain Ready Probes reagent, R37605, Invitrogen-Thermo Fisher Scientific, USA) for nuclei staining, as well as phalloidin (Thermo Fisher Scientific, Waltham, MA, USA) to visualize the actin filaments, overnight at 4 °C [21].

Confocal images were acquired using a Leica SP8 confocal microscope (Leica Microsystems, Milano, Italy). Images were collected through a HC PL FLUOTAR 20×/0.55 DRY objective using sequential acquisitions of the green (excitation 488 nm, emission 493–523 nm), red (excitation 551 nm, emission 557–586 nm) and blue (excitation 405 nm, emission 435–460 nm) channels and the system-optimized pixel size (XY  =  0.18 µm, Z  =  2 µm), as previously described [22]. Images were prepared for publication using Fiji software 1.54p and are shown as the maximum intensity projection along the *z*-axis.

Supplementary: Fragments of tissues from GC and adjacent HM were fixed with 10% formalin for 4 days, rehydrated in deionized H_2_O for 24 h, and then permeabilized with 0.1% Tween20 in PBS. Then, they were blocked in 2% BSA and 0.1% Triton-X-100 in PBS and incubated with CMG for 48 h. After 3 washes in deionized H_2_O, images were acquired using FireFly^®^ technology with the DaVinci Xi camera (Intuitive, Oxford, UK).

### 2.6. CMG Antibody Generation by CEACAM5 Antibody Conjugation

To generate the CMG fluorescent antibody probe, a primary monoclonal anti-CEACAM5 antibody (initial concentration: 0.21 µg/µL) was conjugated with an NIR fluorescent dye using the Mix-n-Stain™ CF^®^800 Dye Antibody Labeling Kit (Biotium, Fremont, CA, USA), following the manufacturer’s protocol.

Briefly, the antibody solution was first concentrated by ultrafiltration via centrifugation at 14,000× *g* for 10 min at RT, using the spin filters provided in the kit. This step also served to buffer-exchange the antibody into a labeling-compatible formulation.

The concentrated antibody was then incubated with the CF^®^800 dye for 15 min at RT to facilitate covalent conjugation. Following the reaction, the labeled antibody was diluted in the provided storage buffer to stabilize the conjugate.

The final concentration of the fluorescently labeled anti-CEACAM5 antibody was 0.173 µg/µL. The conjugated antibody was stored at 4 °C and protected from light until further use in imaging experiments.

### 2.7. In Silico Analysis

The mRNA expression levels of CLDN4 and CEACAM5 in gastric adenocarcinoma samples from the STAD cohort (n = 408) and normal stomach samples (pooled TCGA and GTEX samples, n = 211) were plotted through the box plot function of the GEPIA2 web server [23].

### 2.8. Statistical Analysis

Data analyses were performed using GraphPad Prism Version 8.3.0 for Windows (GraphPad Software, San Diego, CA, USA) or SPSS version 27.0 (SPSS Inc., Chicago, IL, USA). Results are expressed as the mean ± SE (standard error), as detailed in the figure legends. Comparisons of means between two groups of data were performed using the Student’s *t*-test, and multiple comparisons using a one-way ANOVA followed by Dunnett’s post hoc test, with *p* < 0.05 taken to be statistically significant.

## 3. Results

### 3.1. Study Population

Thirty GC patients affected by adenocarcinoma who underwent a gastrectomy at the Azienda Ospedaliero Universitaria Careggi (AOUC) in Florence were enrolled in this study. We excluded patients with distant metastases or pre- or intraoperative T4 lesions (i.e., local invasion of other organs such as the pancreas, spleen, or peritoneum).

The mean age of GC patients (n = 30; twenty-three males, seven females) was 74.5 years (57–88).

The number of patients who underwent perioperative chemotherapy was 18 (60%), while 12 (40%) did not.

Tumor staging was assessed according to the eighth TNM edition. The percentage of stage I (47%) patients was higher compared with the percentage of stage II (30%) and stage III (23%) patients. The clinical/demographic characteristics of GC patients are summarized in Table 1.

The use of human samples was approved by the Local Ethics Committee of University Hospital, Azienda Ospedaliero-Universitaria Careggi, Florence, Italy (n. 27004_bio), according to the Helsinki Declaration, and informed consent was obtained from each patient enrolled in this study.

### 3.2. Identification of GC-Associated Surface Biomarkers

To identify suitable membrane biomarkers for targeted fluorescent imaging in GC, we conducted an extensive literature review focused on molecules with established or emerging roles in GC pathology. This analysis led to the selection of five potential candidate biomarkers based on their reported overexpression in gastric tumors and their membrane localization: carcinoembryonic antigen-related cell adhesion molecule 5 (CEACAM5) [24,25,26], polymeric immunoglobulin receptor (PIGR) [27,28], epithelial cell adhesion molecule (EpCAM) [26,29], olfactomedin4 (OLFM4) [30,31,32], and Claudin4 (CLDN4) [26,33,34]. These markers were prioritized for further evaluation based on their potential usefulness in enhancing tumor-specific signals during intraoperative fluorescence-guided surgery.

**Figure 1:**
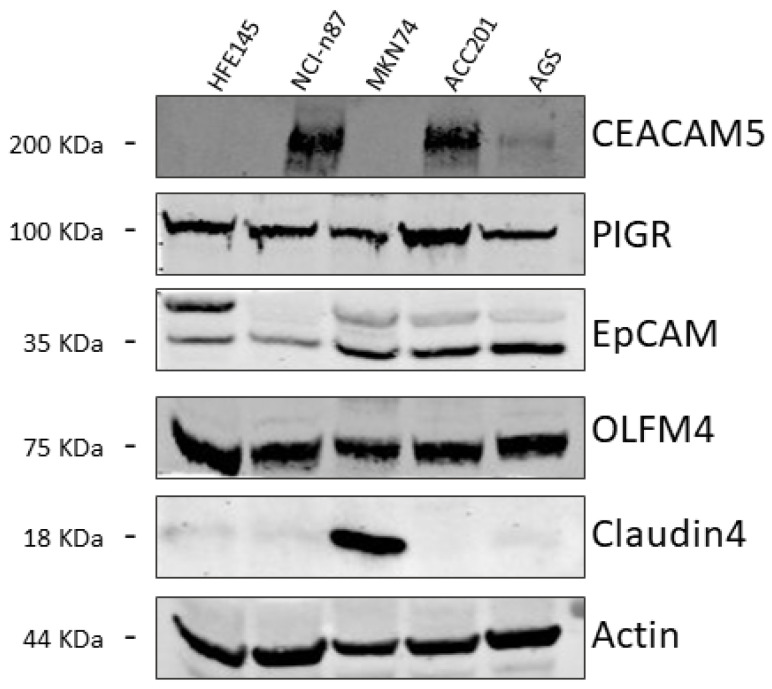
Representative Western blot images of CEACAM5, PIGR, EpCAM, OLFM4, and CLDN4 expression levels in the metastatic NCI-n87 and MKN74 and non-metastatic ACC201 and AGS GC cell lines compared with the healthy gastric mucosa HFE145 cell line. An actin immunoblot was used as a loading control.

Using Western blotting, we first validated the expression of these biomarkers on metastatic (NCI-n87 and MKN74) and non-metastatic (ACC201 and AGS) GC cell lines compared with the healthy gastric mucosa cell line (HFE145) used as the control. Both non-malignant and metastatic cell lines expressed the selected biomarkers. However, only CEACAM5 and CLDN4 were absent in the healthy gastric cell line used as a control, prompting us to focus on these two biomarkers (Figure 1).

**Figure 2:**
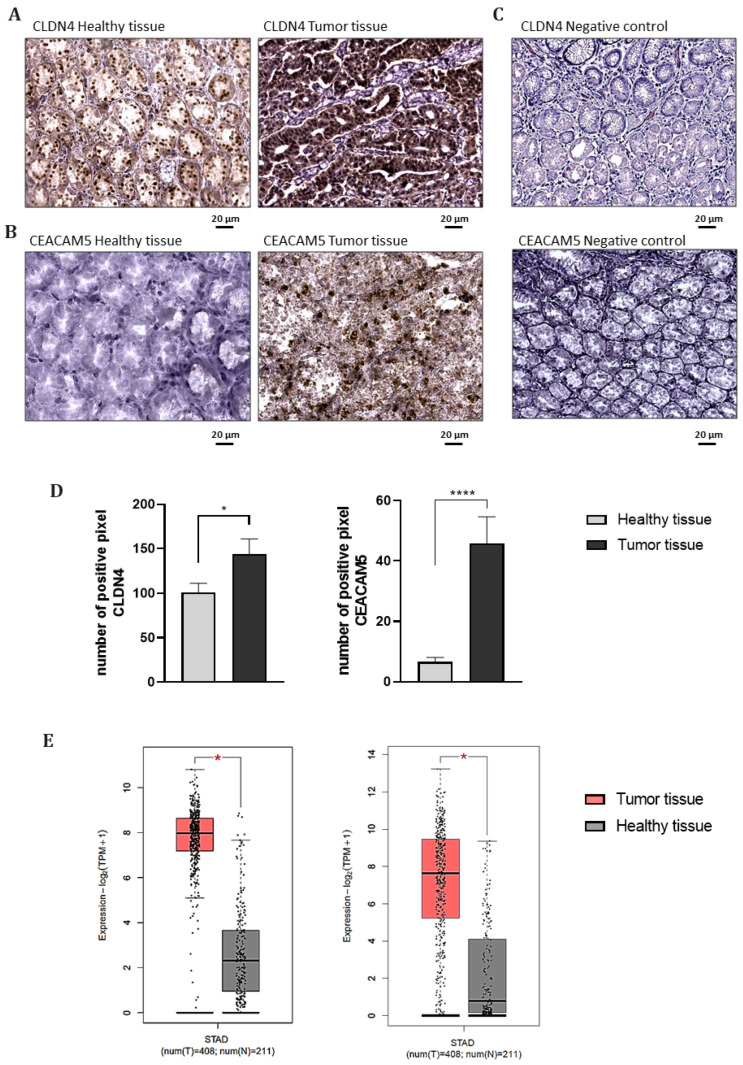
Representative images of immunohistochemical staining of (**A**) CLDN4 and (**B**) CEACAM5 expression on healthy mucosa or tumor tissue sections. Staining with a secondary antibody only was performed for the negative control (**C**). In the bar graphs (**D**), the densitometric analysis of the positive pixels for CLDN4 and CEACAM5 signals is represented. The brown color in the pictures corresponds to the positive signal of the antigen (CLDN4 and CEACAM5). The results are expressed as the mean expression in tumor slices (dark gray bars) compared to healthy mucosa slices (light gray bars) ± SEM. * *p* ≤ 0.05, **** *p* ≤ 0.0001. (**E**) mRNA expression levels of CLDN4 and CEACAM5 in GC samples (grey bars) from the STAD cohort (n = 408) and HM (red bars) (pooled TCGA and GTEX samples, n = 211), plotted using the GEPIA2 web server. * *p* < 0.05.

Using IHC, CLDN4 and CEACAM5 were then evaluated on tumor and healthy tissue sections prepared from paraffin-embedded samples collected from patients who underwent a partial gastrectomy at the Digestive System Surgery Unit, AOU Careggi, Florence (panel A and B in Figure 2, respectively). Secondary antibody-only controls were performed to assess the specificity of the primary antibody staining. The absence of signals in these control sections confirmed that the immunoreactivity observed in the samples was specific to the primary antibody (Figure 2C). Analysis of the positive pixel was carried out to quantify the signals associated with CLDN4 and CEACAM5, revealing that CLDN4 expression was significantly higher in tumor samples compared to surrounding healthy tissues from the same patients (*p* < 0.5). However, its strong expression in healthy tissues from the same patients limited its capacity to effectively distinguish between tumor and healthy tissues (Figure 2D). In contrast, CEACAM5 exhibited significantly higher expression in GC tissues, while its expression in HM was minimal or undetectable (*p* < 0.001) (Figure 2D).

Consistently, CLDN4 and CEACAM5 mRNA levels are significantly higher in gastric adenocarcinomas compared to normal gastric samples from the large TCGA and GTEx cohorts, with CLDN4 showing higher expression levels than CEACAM5 in normal tissues (Figure 2E).

**Figure 3:**
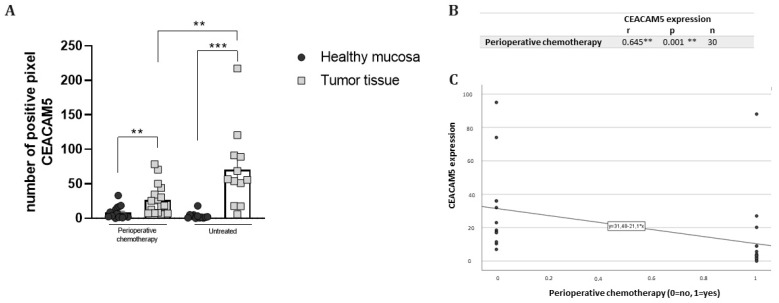
(**A**) Quantification of CEACAM5 expression in our patient cohort, divided into those who underwent perioperative chemotherapy (perioperative chemotherapy) and those who did not (untreated). The results are expressed as the mean expression in tumor slices (dark gray points) compared to healthy mucosa slices (light gray squares) ± SEM. ** *p* ≤ 0.01, *** *p* ≤ 0.001. (**B**) Negative association between CEACAM5 expression levels (ratio of positive pixels in tumor tissue relative to healthy mucosa from the same patient) and perioperative chemotherapy treatment. n = total number of patients included in the analysis. Correlation coefficients (r) and significance levels (P) were calculated using the Spearman method. ** indicates a significant correlation. (**C**) Correlation between CEACAM5 expression (ratio of positive pixels in tumor tissue relative to healthy mucosa from the same patient) and the administration of perioperative chemotherapy (0 = no; 1 = yes). Each point represents a tested sample.

We divided our cohort of patients according to whether or not they had received perioperative chemotherapy. As shown in Figure 3, we observed significantly different CEACAM5 expression patterns between the two groups. Specifically, in the group undergoing perioperative chemotherapy, there was a significant difference in CEACAM5 expression between tumor tissue and HM (*p* < 0.01), but this difference was even more significant in patients who did not receive perioperative chemotherapy (*p* < 0.001). In addition, when comparing tumor tissues, patients who did not receive perioperative chemotherapy showed significantly higher CEACAM5 expression than those who received perioperative chemotherapy (*p* < 0.01), suggesting the potential of future personalized surgery (Figure 3A). To further support these findings, Figure 3 (panel B) shows a significant negative association, assessed by Spearman analysis, between CEACAM5 expression levels (measured as the ratio of positive pixels in tumor tissue relative to HM from the same patient) and perioperative chemotherapy treatment (*p* < 0.001). This result demonstrates that CEACAM5 levels decrease with the administration of perioperative chemotherapy. In addition, the regression line (Figure 3C) depicts the correlation between CEACAM5 expression and perioperative chemotherapy administration (0 = no; 1 = yes). When perioperative chemotherapy is administered, CEACAM5 expression tends to decrease.

We further assessed CEACAM5 levels in male versus female patients and found no significant difference between the groups, implying that CEACAM5 expression does not depend on the patient’s sex (Supplementary Figure S1A). Moreover, we stratified the patients based on TNM stage and compared CEACAM5 expression across the different groups. We observed a trend of increasing CEACAM5 expression with advancing tumor stage, although the difference was not statistically significant (Supplementary Figure S1B).

### 3.3. Assessment of CEACAM5 as a Biomarker

**Figure 4:**
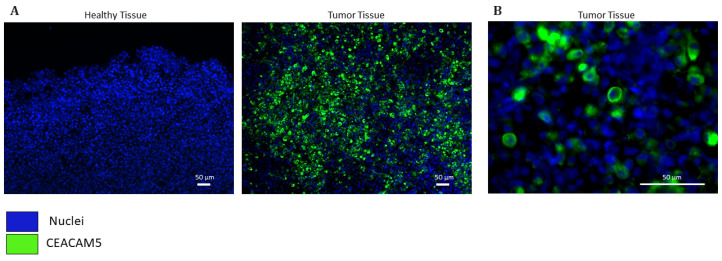
Representative images of CEACAM5 immunofluorescence staining at 10× (**A**) and 40× (**B**) magnifications. GC tissue and healthy adjacent mucosa were labeled by CEACAM5 (green) to highlight membrane localization. The nuclei were indicated by the blue color (NucBlue).

To further investigate CEACAM5 as a candidate for targeted imaging, we evaluated its expression and subcellular localization by immunofluorescence on paraffin-embedded tissue sections from GC patients who underwent a partial gastrectomy at the Digestive System Surgery Unit, AOU Careggi, Florence. Immunofluorescence analysis confirmed robust CEACAM5 expression in GC tissues, while no signal was detected in the adjacent HM (Figure 4A), underscoring its strong potential to discriminate between malignant and non-malignant tissues. High-magnification imaging (40×) revealed that CEACAM5 was predominantly localized at the cell membrane (Figure 4B), a key feature for its applicability in in vivo fluorescence-guided imaging strategies. Notably, tissue sections were excited using an 800 nm wavelength light source, which falls within the NIR range and closely mimics the optical conditions of clinical intraoperative imaging, further supporting the translational relevance of our findings.

**Figure 5:**
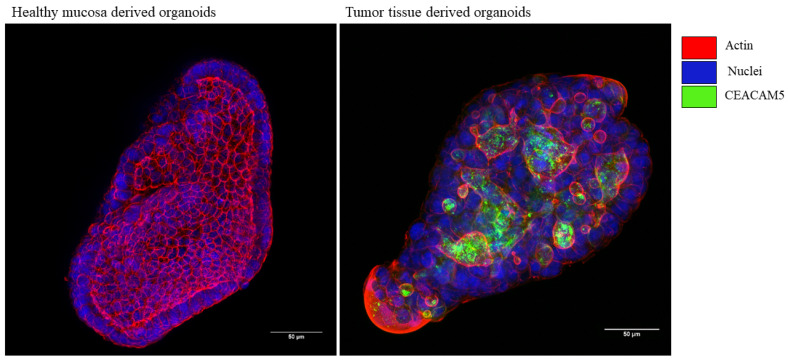
Characterization of CEACAM5 expression on 3D organoids using confocal microscopy. Organoids were labeled with an anti-CEACAM5 antibody (in green), with phalloidin fluorescein isothiocyanate to visualize actin filaments (in red), and with NucBlue for nuclei staining (in blue). Confocal images were acquired with a Leica SP8 confocal microscope, processed using Fiji software, and shown as the maximum intensity projection along the *z*-axis.

Next, we established an organoid model using processed samples obtained from the HM and GC tumor tissue of patients who underwent a gastrectomy. Confocal microscopy visualization of 3D images enables the precise assessment of biomarker staining on the surface of cancer cells within the microenvironment, thus improving the effectiveness and efficiency of identifying promising biomarkers such as CEACAM5. We confirmed that CEACAM5 expression was localized in organoids derived from tumor tissue and not, or to a lesser extent, in organoids derived from healthy gastric tissue (Figure 5). Moreover, our organoids are good models for biomarker assessment because they resemble the characteristics of the organ of origin.

### 3.4. CEACAM5 Antibody Conjugation and Validation

Next, we prepared a fluorescently labeled anti-CEACAM5 antibody through conjugation with an activated and commercially available fluorescent molecule that is spectrally similar to ICG. The resulting molecule was an antibody with an excitation wavelength of 800 nm, which we named CMG. This conjugate was designed for potential future in vivo applications.

**Figure 6:**
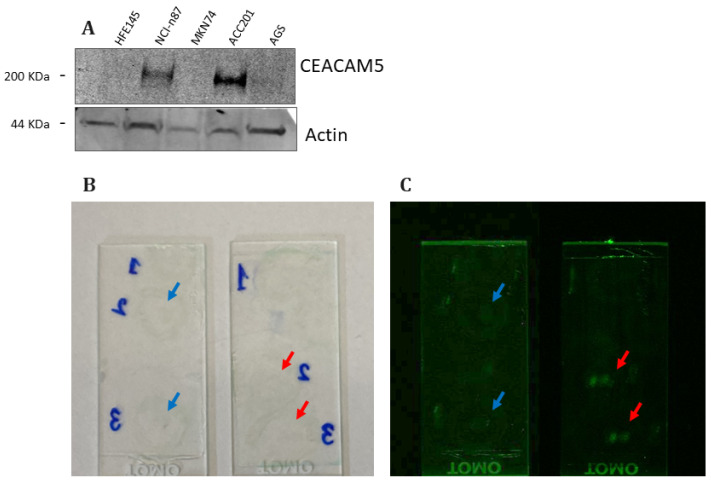
(**A**) Representative Western blot images of CEACAM5 expression levels as detected by the CMG antibody in the metastatic NCI-n87 and MKN74 and non-metastatic ACC201 and AGS GC cell lines compared with the healthy gastric mucosa HFE145 cell line. An actin immunoblot was used as a loading control. Representative images of CEACAM5 immunofluorescence staining in healthy (blue arrows) and tumor (red arrows) tissues derived from the same patient. (**B**) Brightfield images of stained tissue sections. (**C**) Near-infrared (NIR) fluorescence images acquired at 800 nm using the Odyssey Infrared Imaging System. Tissue slices were labeled with the CMG antibody to evaluate its binding specificity for tumor tissue.

**Figure 7:**
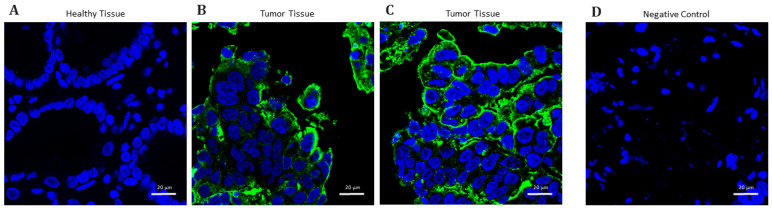
Representative images of CEACAM5 immunofluorescence staining at a magnification of 63×. Healthy tissue (**A**) and tumor tissues were labeled by anti-CEACAM5 (green) antibodies, (**B**) the CMG fluorescent antibody, or (**C**) a commercial primary antibody used as a control to assess the binding specificity of the CMG antibody. (**D**) The secondary antibody on its own was used as a control for primary antibody-binding specificity. The nuclei are indicated by the blue color (NucBlue).

We evaluated the binding capacity of the CMG antibody to ensure that the conjugation reaction did not compromise its specificity and affinity. To accomplish this, we first evaluated its binding affinity to previously utilized GC cell lines, demonstrating that CMG effectively highlighted CEACAM5 expression with a signal intensity comparable to that achieved using a primary and secondary antibody combination (Figure 6A). Notably, the signal was missing in the healthy gastric cell line HFE145. Next, we conducted immunofluorescence staining on tissue sections obtained from patients who had undergone a partial gastrectomy at the Digestive System Surgery Unit, AOU Careggi, Florence. Compared to brightfield images of tissue slices (Figure 6B), labeled tissue slices observed at 800 nm exhibited a positive signal only in tumor samples, without a detectable signal in the corresponding HM (Figure 6C). Furthermore, in comparison with healthy tissue (Figure 7A), the tumor samples (Figure 7B) labeled with a CMG antibody displayed a distinct membrane-associated signal, comparable to that observed with the commercial anti-CEACAM5 antibody that lacks NIR conjugation (Figure 7C).

Finally, to evaluate CMG’s compatibility with the DaVinci robotic surgical platform, tissue fragments were stained with the fluorescently labeled CMG antibody and visualized using the FireFly^®^ fluorescence imaging camera. The FireFly^®^ system successfully detected the signal in the labeled tissue fragments (Supplementary Figure S2). Notably, the signal was significantly more intense in tumor tissue than in HM, supporting both the specificity of CMG and visibility under surgical imaging conditions. Overall, these results confirm that the antibody retained its specificity even after conjugation.

## 4. Discussion

The tumor-targeted fluorescent molecular imaging approach has the potential to address key challenges in the intraoperative visualization of metastatic lymph nodes and the assessment of resection margins in GC.

Indeed, achieving a radical resection remains a significant intraoperative challenge. Studies have shown that microscopically tumor-positive resection margins (R1 resection) are still detected in approximately 7% of GC patients. This is responsible for higher rates of peritoneal recurrence and poorer overall survival [35,36].

Another relevant challenge in GC surgery management is sentinel lymph node (SLN) mapping. Growing interest in fluorescent dyes, especially the peritumoral injection of indocyanine green (ICG), stems from their proven ability to identify complete lymphatic drainage patterns, including aberrant flow. However, refining this approach to selectively highlight only the SLN would further enhance surgical precision and clinical outcomes. Achieving this goal relies on the careful selection and application of molecular imaging targets to ensure optimal tumor visualization.

In this study, we first aimed to identify a promising tumor-specific target for GC. Among the five candidates analyzed, CEACAM5 emerged as a highly expressed biomarker in primary GC tissue specimens, with positive expression observed in more than 85% of cases.

Although EpCAM, PIGR, OLFM4, and CLDN4 have been previously identified as membrane markers for GC, they did not effectively discriminate between HM and tumor tissue in our patient cohort. Compared to previous reports, these discrepancies may be attributed to differences in scoring systems, primary antibodies, or antigen retrieval techniques used in IHC staining. Additionally, factors such as inter- and intratumoral heterogeneity and the difference in the sample sizes of previous IHC studies may have contributed to the variation in the outcomes [37].

Consistent with our findings, high CEACAM5 expression in GC tissues was reported, together with a good discrimination capacity between HM and tumor tissues [16,38]. Moreover, our in silico analysis of mRNA expression levels of CLDN4 and CEACAM5 using data from the STAD cohort revealed consistent results across a large group of patients.

This discriminatory ability was preserved in the patient-derived organoid model, further validating CEACAM5 as a robust tumor-specific biomarker. The development of organoids has coincided with the rise of advanced volumetric imaging techniques capable of characterizing the 3D architecture of whole-mount tissues. Compared to traditional 2D imaging methods based on tissue sectioning, 3D imaging offers a powerful platform for studying biomarker distribution in a spatially preserved, physiologically relevant context [39]. These enhanced capabilities are crucial for a comprehensive understanding of cellular composition, morphology, fate decisions, and cell–cell interactions within biological samples [40,41].

Building on these results, the subsequent aim of our study was to develop a novel anti-CEACAM5 antibody conjugated with an NIR fluorophore for real-time tumor tissue identification during surgical procedures, which we designated CMG. We further evaluated its binding specificity to assess its potential for future clinical application.

We initially evaluated the CMG fluorescent antibody on GC cell lines using Western blotting. The fluorescent signal obtained with CMG was comparable to that observed using a commercial primary antibody followed by a fluorescently labeled secondary antibody. However, CMG’s signal intensity was slightly lower, probably due to the absence of signal amplification that occurs with secondary antibody binding. Indeed, since CMG is directly labeled with a fluorescent dye, it provides more specific but slightly less intense staining than the two-step amplification method. To confirm the presence of a specific and positive signal in tumor tissues at the 800 nm wavelength, we used the Odyssey Infrared Imaging System. This analysis revealed a fluorescent signal only in tumor slices, while no signal was detected in the adjacent HM, indicating that CMG specifically binds to tumor tissue. After that, to deeply observe the tissues at higher magnifications at a cellular level, due to the absence of a microscope capable of excitation at 800 nm and detection at 820 nm, we employed an alternative approach. To visualize CMG, we used a secondary AlexaFluor-conjugated antibody, allowing us to compare its signal with that of a commercially available primary and secondary antibody combination. To ensure that CMG could be effectively detected using the DaVinci surgical system, we stained tissue fragments with the fluorescently labeled CMG antibody and visualized them using the FireFly^®^ fluorescence imaging camera. The system successfully distinguished the fluorescent signal, confirming the feasibility of detection. Notably, the fluorescence intensity was higher in tumor tissues compared to HM, highlighting the specificity and potential clinical applicability of CMG. Among fluorescent molecules targeting GC for precision surgical purposes that are under preclinical and clinical investigation, SGM-101 is a novel NIR imaging agent that combines the BM104 dye with a chimerized monoclonal antibody targeting the carcinoembryonic antigen (CEA) and that is fluorescent at 705 nm. This agent has demonstrated promising results across various murine models of digestive cancers, including orthotopic tumors, peritoneal carcinomatosis, and hepatic metastases [42]. Currently, SGM-101 is undergoing clinical validation for colorectal, pancreatic, and lung cancers, while other studies are underway to assess its preclinical efficacy in GC [43,44]. Cox and colleagues developed a humanized anti-CEA antibody (M5A) conjugated with the NIR dye IRDye800CW that is specific for GC. Their approach demonstrated efficacy in orthotopic mouse models and patient-derived orthotopic xenograft (PDOX) models, where tumor fragments from GC samples were implanted into the stomachs of mice [45,46]. Compared to previous molecules, our antibody is conjugated to a distinct fluorochrome that fluoresces at 800 nm, aligning optimally with the NIR spectrum range of the Da Vinci Surgical System and laparoscopic columns equipped for NIR detection.

Our data revealed a significant difference in CEACAM5 expression between patients who underwent perioperative chemotherapy and those who did not. In patients who did not undergo perioperative chemotherapy, CEACAM5 expression was better preserved within the tumor, facilitating its detection. Conversely, in patients who underwent perioperative chemotherapy, the tumor tissue appeared largely decellularized and more fibrotic, leading to a considerable loss of CEACAM5 expression, although the difference between the tumor and HM derived from the same patients was still significant. This suggests that the probe would be most effective in patients with untreated tumors, where it can provide clearer and more reliable tumor visualization. Therefore, for potential future clinical applications, we believe the probe may be recommended for those patients who have not undergone perioperative chemotherapy. These data foresee the possibility of implementing personalized treatments, allowing clinicians to determine the most appropriate intervention type for each patient based on a set of individualized criteria. Notably, even in patients who received perioperative chemotherapy, CEACAM5 was strongly expressed in GC tissue but not in HM, allowing for clear discrimination between the two.

In addition, we evaluated whether CEACAM5 expression differed between male and female patients with GC. No statistically significant difference was observed between the two groups, suggesting that CEACAM5 expression may indeed be sex-independent. However, because the female cohort was substantially smaller than the male cohort, this analysis may have been too underpowered to detect a true difference. In addition, the analysis of CEACAM5 expression in patients stratified by TNM stage revealed a progressive increase in CEACAM5 levels corresponding to advancing tumor stage. Although the differences did not reach statistical significance, this trend suggests that CEACAM5 expression tends to be higher in more advanced tumors, potentially reflecting its involvement in tumor progression or aggressiveness.

Prior to clinical evaluation, the CMG probe will also undergo in vivo testing and validation to lay the foundation for its clinical applicability, ensuring its effectiveness for future implementation in targeted surgery.

## 5. Conclusions

In this study, we developed a fluorescent molecular imaging probe specifically designed for GC, with the aim of integrating it into surgical imaging platforms for the real-time visualization of cancerous tissues during surgery. By enhancing tumor delineation, our new probe holds the potential to significantly improve the precision of fluorescence-guided surgery, reducing residual disease and ultimately enhancing patient outcomes. To ensure its clinical applicability, the probe will undergo further in vivo testing and validation, rigorously assessing its safety for future implementation in targeted surgical procedures. Furthermore, the integration of such molecular imaging tools into standard surgical workflows holds promise for a new paradigm in precision oncologic surgery, with broader applications across other CEACAM5-expressing malignancies.

## Figures and Tables

**Table 1:** Summary of the characteristics of GC patients enrolled in this study.

	Patients
Number	30
Age	74.5 (57–88)
Gender	
Male	23 (77%)
Female	7 (23%)
Perioperative	
chemotherapy	
Yes	18 (60%)
No	12 (40%)
TNM stage	
I	14 (47%)
II	9 (30%)
III	7 (23%)

## Data Availability

All data generated or analyzed during this study are included in this published article [and its Supplementary Information Files]. The authors declare their compliance with the digital image and integrity policies.

## Supplementary Materials

The following supporting information can be downloaded at: https://www.mdpi.com/article/10.3390/biomedicines13081812/s1, Figure S1: Evaluation of CEACAM5 tissues expression. (A) Representation of CEACAM5 expression in our patient cohort, divided into males and females. (B) CEACAM5 expression in groups of patients divided by the tumor progression stadium (I, II or III based on TNM tumor stage. The results are expressed as mean expression in tumor slices (dark gray points) compared to healthy mucosa slices (light gray squares) ± SEM. * *p* ≤ 0.05, ** *p* ≤ 0.01, *** *p* ≤ 0.001; Figure S2: Representative images of CMG antibody staining visualized at the Da Vinci robotic platform. Healthy tissue (A,B) Tumor tissue were labeled by CMG antibody.
