# Supplementary figures and images for: Targeting CEACAM5: Biomarker Characterization and Fluorescent Probe Labeling for Image-Guided Gastric Cancer Surgery
Source: Biomedicines. 2025 Jul 24;13(8):1812. doi: 10.3390/biomedicines13081812 (PMC12383877; doi:10.3390/biomedicines13081812)

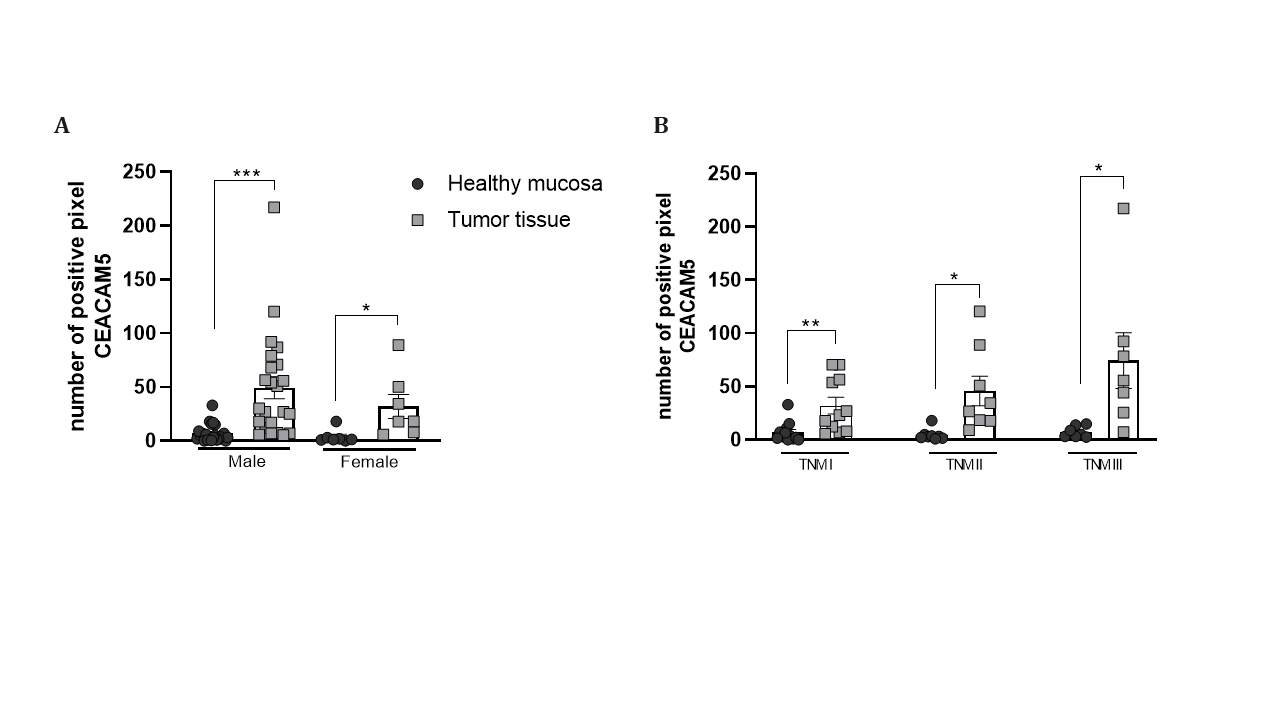

Supplement: Supplementary file 1 [file biomedicines-13-01812-s001.zip › Supplementary Figure S1.tif]

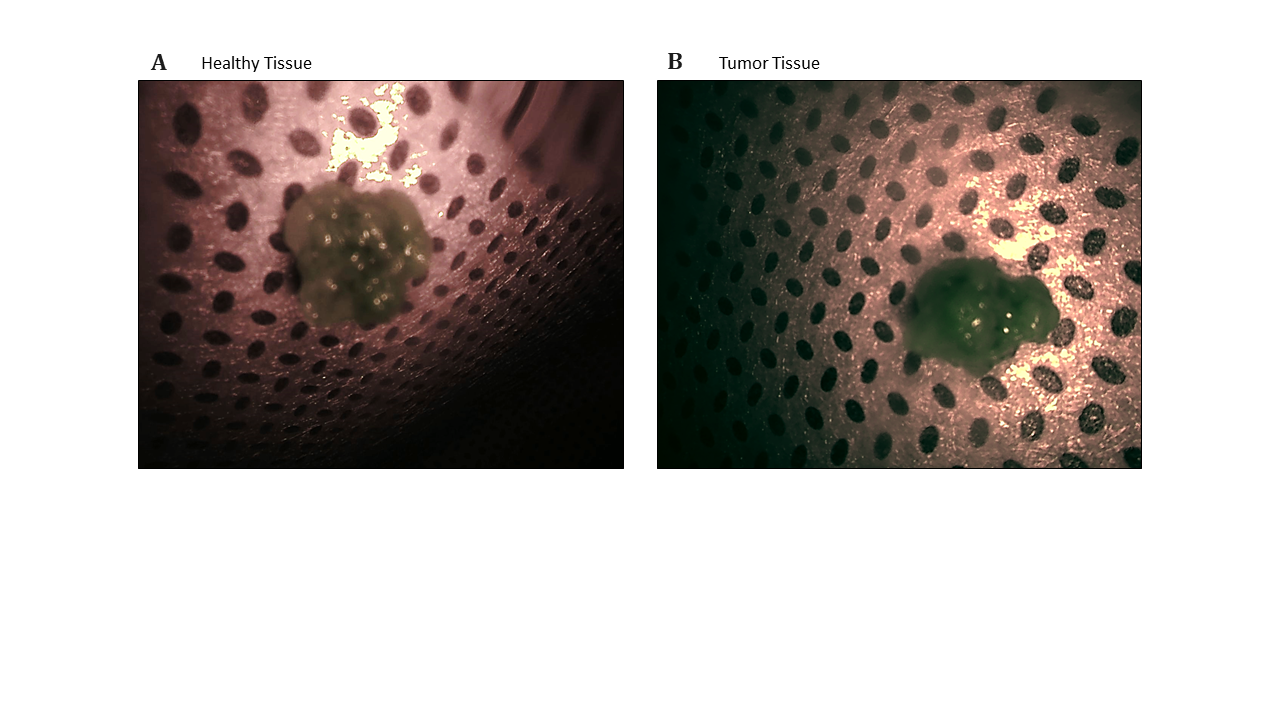

Supplement: Supplementary file 1 [file biomedicines-13-01812-s001.zip › Supplementary Figure S2.tif]
